# Protein spot arrays on graphene oxide coatings for efficient single-cell capture

**DOI:** 10.1038/s41598-022-06225-4

**Published:** 2022-03-10

**Authors:** R. Kumar, S. Llewellyn, S. K. Vasantham, Kaiwen Nie, S. Sekula-Neuner, A. Vijayaraghavan, M. Hirtz

**Affiliations:** 1 Institute of Nanotechnology (INT) and Karlsruhe Nano Micro Facility (KNMF), Karlsruhe Institute of Technology (KIT), Karlsruhe, Germany; 2 Department of Materials, The University of Manchester, Manchester, UK; 3 Blond McIndoe Laboratories, Faculty of Biology Medicine and Health, University of Manchester, Manchester, UK; 4 n.able GmbH, Eggenstein-Leopoldshafen, Germany

**Keywords:** Biomedical engineering, Graphene

## Abstract

Biomedical applications such as cell screening or cell–cell interaction studies require placement and adhesion of cells on surfaces with controlled numbers and location. In particular, single-cell arraying and positioning has come into focus as a basis of such applications. An ideal substrate would combine biocompatibility with favorable attributes such as pattern stability and easy processing. Here, we present a simple yet effective approach to single-cell arraying based on a graphene oxide (GO) surface carrying protein (fibronectin) microarrays to define cell adhesion points. These capture NIH-3T3 cells, resulting in cell arrays, which are benchmarked against analogous arrays on silanized glass samples. We reveal significant improvement in cell-capture performance by the GO coating with regards to overall cell adhesion and single-cell feature occupancy. This overall improvement of cell-arraying combined with retained transparency of substrate for microscopy and good biocompatibility makes this graphene-based approach attractive for single-cell experiments.

## Introduction

Single- and low-density cell arrays have garnered significant attention for their promising potential in biomedical research where selective and precise cell positioning is key^1,2^. Experiments such as cell–cell communication or artificial cell-network assembly, necessary in drug screening or in vitro biomedical research, require sophisticated single-cell analysis to evaluate overall experimental components^3,4^. Thus, the effective production of single- or few-cell arrays is vital to these efforts. To achieve this, several engineering approaches have been developed to generate suitable cell arrays. These commonly involve designing advanced microwells, followed by targeted cell dispensing e.g. through a microfluidic setup^5,6^. Also, single cells can also be dispensed from a printer or spotter to formulate single-cell arrays^5,7^. Recently, 3D printing has been utilized to even produce 3D tissues by single-cell dispensing^8^. While these methods effectively position cells with high accuracy to produce desired single-cell arrays, they involve complex and costly infrastructure.

An alternative, simpler method to establish single-cell arrays involves engineering substrates used for cell incubation to define spatial-specific cellular adhesion. Examples of this strategy include chemically tuning the material or patterning the substrates with micro-scale protein arrays for cells to preferentially bind onto. The latter approach is possible through various spotting techniques, including printing (inkjet, microcontact) or stamping (polymer pen lithography, capillary spotting) methods. The different spotting techniques vary with respect to their pattern-spacing resolution and individual protein feature sizes. However, each technique has the capability to promote single- or few-cell occupancy in the patterned regions, thereby producing desired cell-arrays^5,9^. Crucially, spotting techniques separate the substrate fabrication step from the cell seeding procedure. This allows for complex array designs within basic lab environments and provides a versatile method where one can modify arrays on the fly in between experiments, through changing pattern designs. Moreover, it permits a variety of substrates or materials as basis for the printing or spotting process, which may be necessary depending on the experimental outcome, or desired application.

Substrates suitable for spotting procedures in cell array production must allow a high density of protein to be deposited and adhere to the surface and maintain protein function^10^. Patterned substrates must also be susceptible to a blocking procedure to inhibit non-specific adhesion and prevent cells from attaching at unwanted areas outside or in-between array features. Furthermore, substrates must exhibit good biocompatibility, without adversely impacting cellular function and promote selective adhesion once patterned. From a fabrication perspective, coated substrates should not significantly impact physical substrate features such as transparency or roughness, nor require special handling for cell culture.

Graphene oxide (GO) is the derivative of graphene which exhibits unique physicochemical properties and has gained promising attention across numerous disciplines, including biomedical research^11–13^. GO has been applied as a culture substrate for a variety of cell populations in vitro and implemented in scaffold materials for tissue engineering^14,15^. GO is a strong candidate for spotting techniques as it has shown compatibility with numerous cell populations, is susceptible to protein functionalization and can be coated onto different carrier substrates without greatly impacting transparency and roughness, potentially making it a universal coating for the spotting procedure on arbitrary underlying substrates. Moreover, GO coatings do not require special handling in cell culture.

Here, we evaluate GO as a substrate suitable for generating single-cell microarrays through assessing protein spotting features and subsequent cell attachment efficiency. Protein micropatterns were generated on GO surfaces with feature sizes applicable for single-cell attachment using the microchannel cantilever spotting (µCS) technique, as µCS has previously shown to be effective in producing arrays of desired dimensions^16,17^. Antibody interactions were used to investigate deposited proteins functionality, alongside cell adhesion experiments to confirm desired, single-cell array resolution. GO based arrays were consequently compared against a silanized glass substrate to assess the material’s effectiveness as basis for single-cell arrays.

## Results

### Spotting of protein arrays

**Figure 1:**
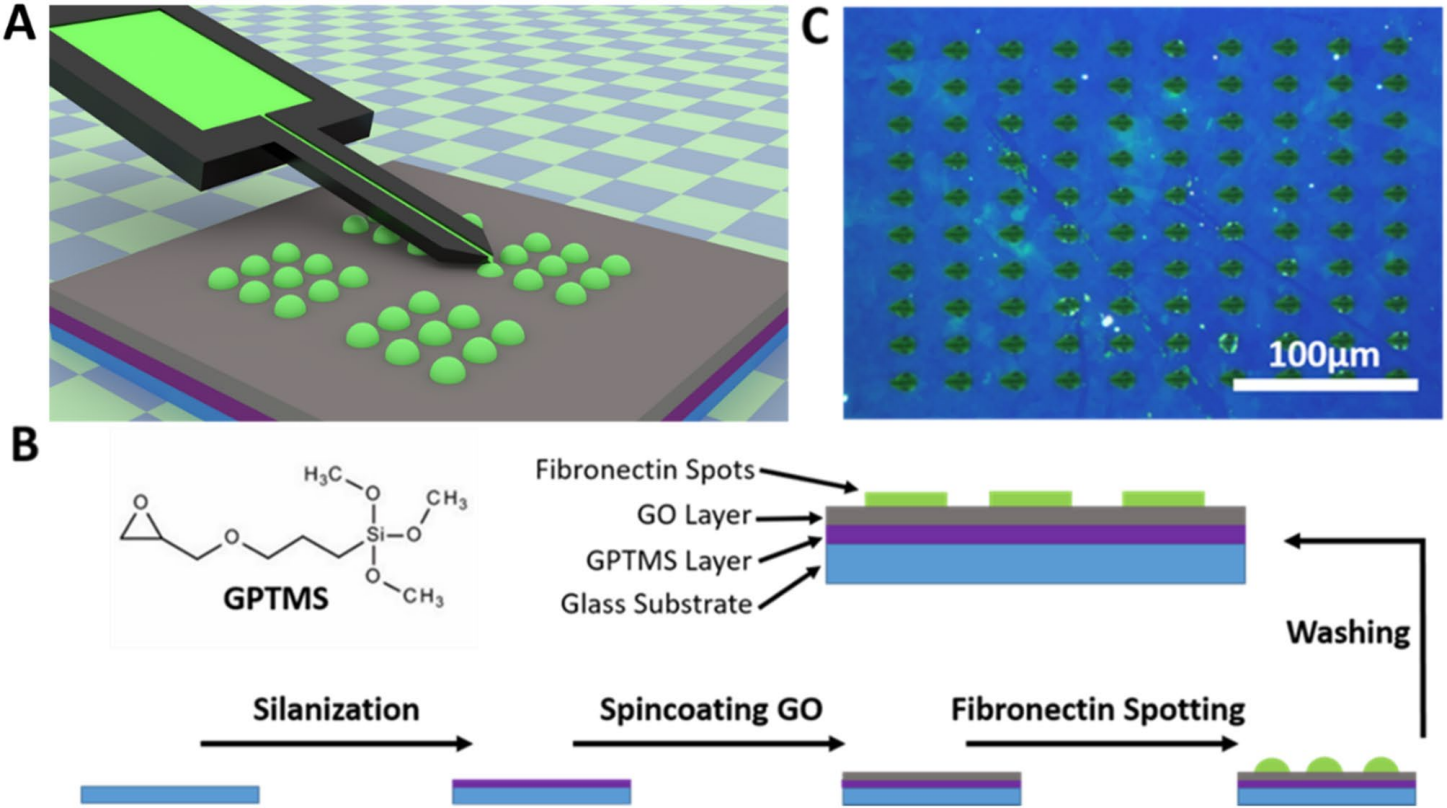
Preparation of protein arrays on graphene oxide substrates. (**A**) Scheme of microchannel cantilever spotting (µCS) technique, used for spotting fibronectin onto graphene oxide substrates. (**B**) Steps in the preparation of the protein arrays: a clean glass substrate is silanized with GPTMS. Next, Graphene oxide (GO) flakes are deposited by spin coating and fibronectin containing ink is spotted by µCS. After washing away excess ink, fibronectin spots remain on the substrate. (**C**) Fibronectin ink droplet microarray under polarized light after spotting (pre-wash).

To elucidate the feasibility of spotting the miniaturized protein patterns onto GO coatings, µCS was used to generate droplet microarrays under controlled environment and parameters. The protein microarray spotting process is illustrated schematically in Fig. 1. To prepare a homogeneous GO layer for spotting, glass substrates were cleaned by sequential sonication in chloroform, isopropanol and water, and then activated using oxygen plasma. The treated glass substrates were silanized with (3-Glycidyloxypropyl)trimethoxysilane (GPTMS). The silanized substrates were spin coated with GO flakes dispersed in DI water. To allow later for visualization of the protein array, fluorescently labelled adhesion protein (CF488A labelled fibronectin) was used for spotting. Fluorescence enables easy quality checks of pattern stability even after excess ink was washed away. The water-based fibronectin ink solution was admixed with 20 vol% glycerol to avoid ink prematurely drying on the spotting tip. To generate protein arrays, the ink-filled spotting tip was brought in contact with GO material, allowing ink to flow from tip to sample by capillary forces. The typical outcome (pre-washing) is shown in Fig. 1C. Here, femtoliter sized droplets deposited via spotting are clearly visible under microscopy with polarized light, where the curvature of the droplets causes a cross-shaped feature visible in each spot. After spotting, the samples were left undisturbed overnight in ambient conditions to allow protein adhesion by physisorption. Excess unadsorbed ink was washed with DI water prior to further experimentation.

**Figure 2:**
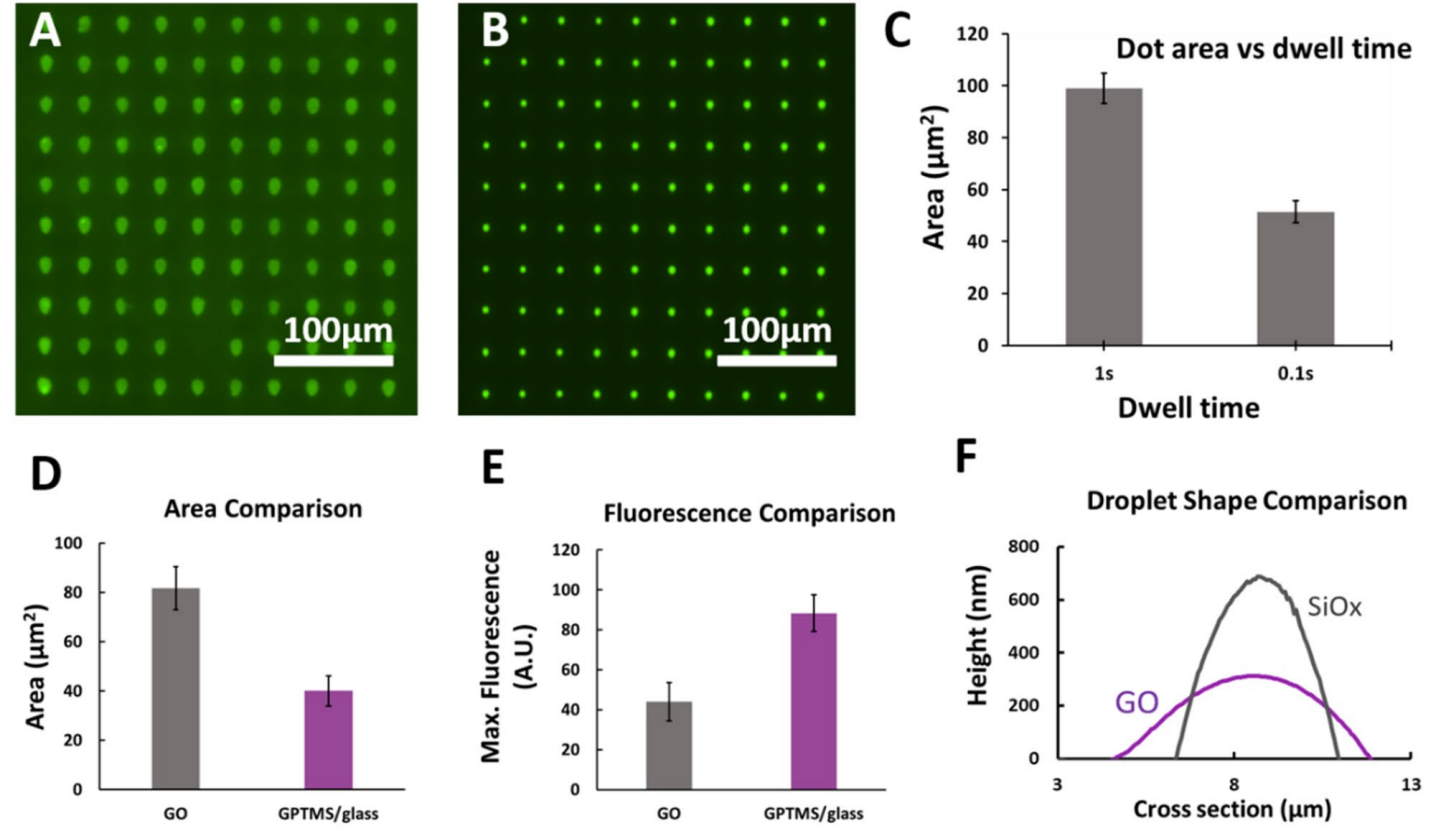
Comparison of protein spotting results on GO and control substrates. Fluorescent microscopy image of a typical spotting outcome on (**A**) a graphene oxide substrate and (**B**) a GPTMS/glass substrate, before removing of excess ink. (**C**) Change in droplet size on graphene oxide substrates for long and short spotting dwell time. (**D**) Comparison of droplet size for GPTMS/glass and graphene oxide substrates from 3 different prints. (**E**) Maximal fluorescence of droplets for GPTMS/glass and graphene oxide substrates from 3 different prints. (**F**) Comparison of averaged AFM profile lines of droplets on a SiO_x_ surface and graphene oxide surface.

Generally, the size of droplets deposited from µCS are significantly influenced by surface hydrophilicity of the substrate and the duration of the tip being in contact with the sample (dwell time)^16,18^. Comparing manufactured protein arrays on GO coatings with those on GPTMS coated glass, which is expected to be less hydrophilic than GO^19^, it is clear that the deposited droplets and resulting array features are significantly larger on the GO coating under similar spotting conditions of 0.1 s dwell time and 30% relative humidity (RH), as shown in Fig. 2A,B. The resulting array size features changed significantly when adjusting the dwell time (Fig. 2C). Slow and fast probing, defined by a dwell time of 1 s and 0.1 s, resulted in droplet areas on GO coatings averaging 98.9 ± 5.7 µm^2^ and 51.4 ± 4.2 µm^2^, respectively.

Quantifying the base area of the droplet features across GO and GPTMS glass surfaces (Fig. 2D) shows 81.7 ± 8.7 µm^2^ versus 40.0 ± 6.2 µm^2^ in agreement to the visual impression of reduced feature size on GPTMS. At the same time, the maximal fluorescence intensity in each feature rises to 88.3 ± 9.2 a.u. compared to 44.0 ± 9.6 a.u. on GO, as shown in Fig. 2E. This can be understood by two mechanisms at play. Firstly, the fluorescent labelling of fibronectin can be partly quenched upon direct contact with the GO, thereby reducing observable fluorescence^20^. On the other hand, due to differences in droplet shape deposited on the substrates, there might be a larger amount of ink and consequently fibronectin deposited on the GPTMS surface.

While excess ink is removed during washing, under similar incubation times more protein may adhere to the GPTMS surface following washing. To exemplify the different droplet shapes, atomic force microscopy (AFM) measurements were compared across droplets deposited on GO and SiO_x_ surfaces (Fig. 2F). Compared to the droplet profile on GO coatings (contact angle from equi-scaled line profile: 6.5° ± 1.8°), the average droplet feature on the more hydrophobic SiO_x_ surface shows a steeper line profile (contact angle from equi-scaled line profile: 38.6 ± 2.6°) but with greater area underneath. This corresponds to features with smaller spread and a larger volume of droplet deposition, respectively. The bigger volume of the deposited droplet sitting on a smaller base area can lead to a higher physisorption of protein on the surface.

### Cell capture on the protein microarrays

**Figure 3:**
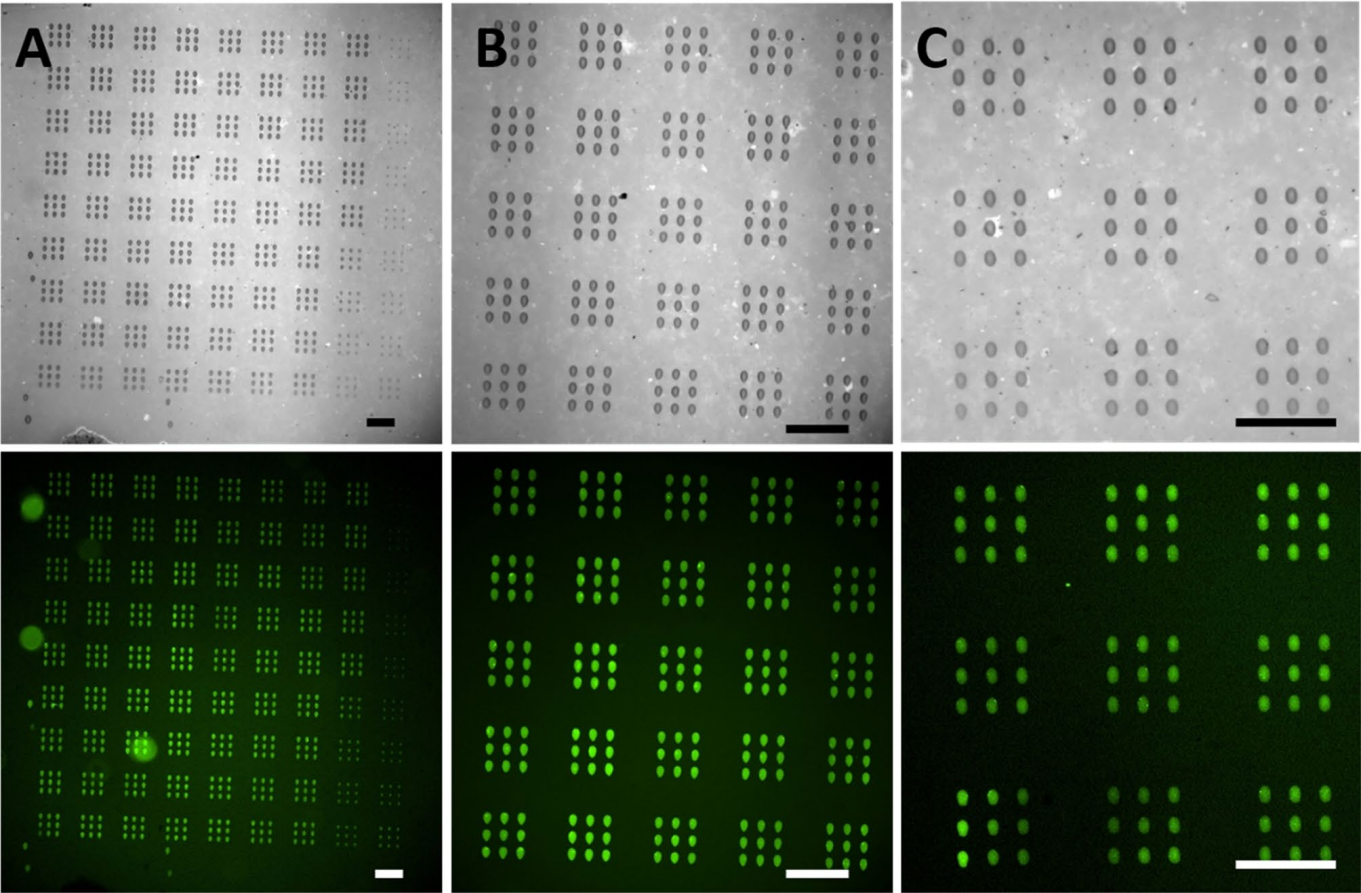
Protein microarrays on GO coatings. (**A**) Bright field and corresponding fluorescence image of a 729 features fibronectin microarray (9 × 9 feature subunits in a 9 × 9 array over an area of 1260 × 1260 µm^2^). (**B**) Subset of the microarray in (**A**). (**C**) Small-area 3 × 3 array of similar subunits in bright field and in fluorescence. All images were taken prior to washing step (to obtain visibility in bright field), scale bars equal 100 µm.

Having established that uniform protein arrays can be produced on GO surfaces, their functionality in generating single-cell arrays was explored. Here, 3 × 3 arrays of dots were spotted as subunit matrix in an array of 9 × 9 subunits, totaling 729 features over an area of 1260 × 1260 µm^2^ (Fig. 3).

**Figure 4:**
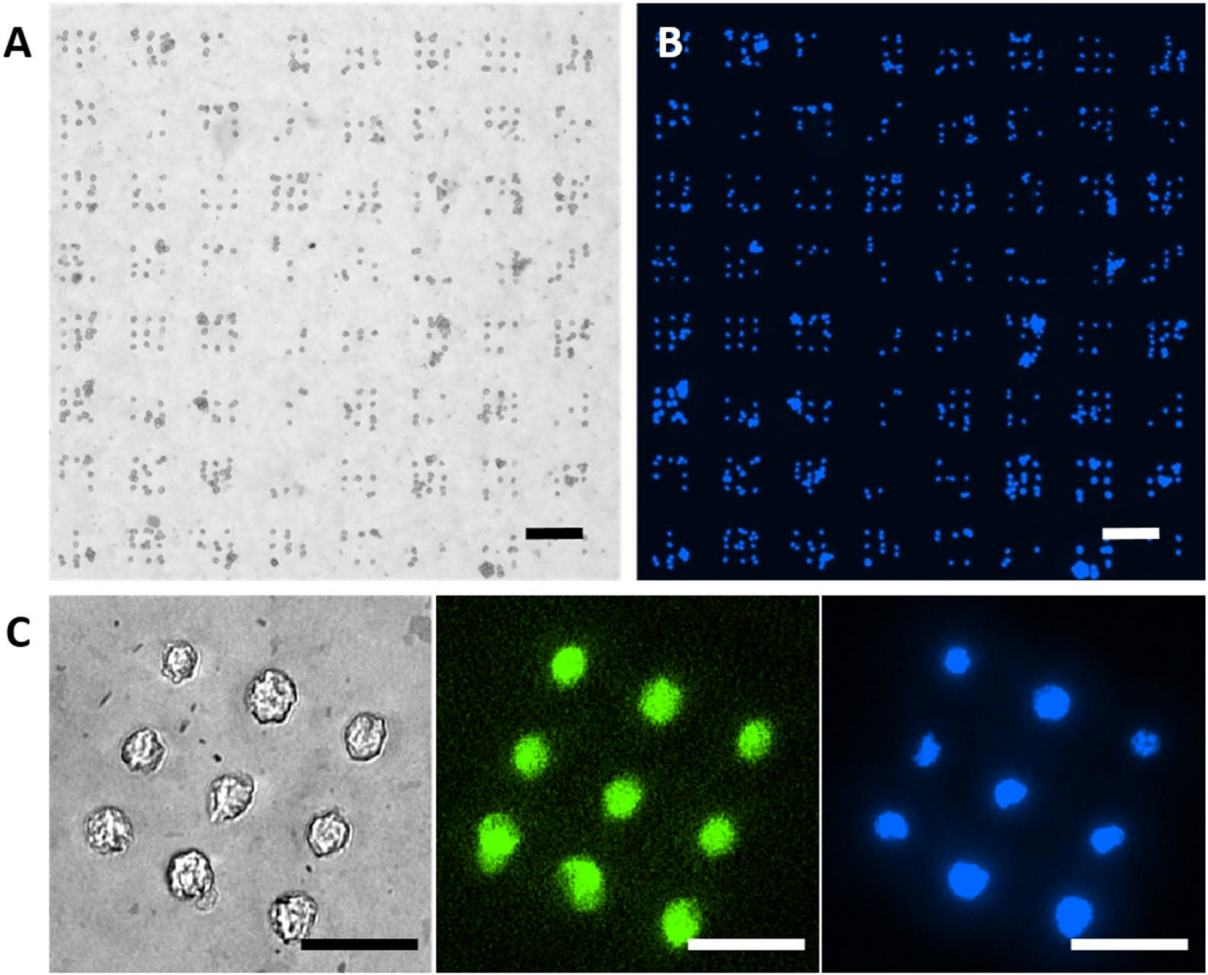
Comparison of cell adhesion on GO coating and control. (**A**) Bright field image of 3T3 cells on a protein microarray spotted on a GO coating. Scale bar equals 100 µm. (**B**) Corresponding fluorescence image of stained cell nuclei. Scale bar equals 100 µm. (**C**) Close-up of a single pattern subunit in bright field, and single fluorescence channels for fibronectin (green) and cell nuclei (blue) signal. Scale bars equal 50 µm.

To test protein functionality, arrays were incubated with fluorescently labelled antibodies against fibronectin. As these antibodies can successfully bind to the protein array, the fibronectin seems accessible and in functional state on the GO coating (Fig. S2). After having established that the protein function is preserved, experiments involving cell attachment were conducted, to elucidate performance towards cell arraying applications. 3T3 fibroblast cells were applied to evaluate adherence, due to their high affinity to fibronectin. Two different incubation strategies were employed to compare surface blocking influence on cell attachment incubated on GO and silanized glass substrates. To confirm this, GO and silanized glass substrates patterned with protein microarrays were either blocked with bovine serum albumin (BSA) prior to cell incubation, which ensures suppression of non-specific attachment^21^, or used as is directly after the patterning process. To assess cell adhesion, cell-nuclei were stained after incubation, enabling easy recognition and counting of individual cells. Figure 4 shows a typical outcome of a cell incubation experiment on a GO sample.

The quantification of cells adhering to the microarray patterns reveal high efficacy of single-cell pattering on GO coatings in comparison to silanized glass control (Table 1).

**Table 1:** Cell adhesion on protein microarrays.

Substrate type	Blocking	Number of cells		Number of features with X cells on it^a^				
		On feature	Off feature	0	1	2	3	> 3
GO coating	–	477	183	339	222	96	21	0
	BSA	915	0	250	255	88	31	28
Silanized glass	–	95	37	650	63	9	4	0
	BSA	220	0	549	103	18	7	9

Quantification of the number of cells adhered onto GO coating versus the silanized glass substrates with and without prior BSA blocking.

^a^Sum of features in the table does not match up to the total features in an array, as of exclusion of the cell-bridged features (see supporting information Fig. S3 for details).

Cells attached to the substrate region between fibronectin array features are classified as ‘off feature’ and the ones on the fibronectin array features as ‘on feature’ (Fig. S3). In general, the number of adhered cells reduces with subsequent washing steps (Fig. S4, Table S2), and while with only one washing step the ‘off feature’ adhesion of cells is still high, already three times washing reveals high alignment between cells and protein array and was chosen for the further array characterization. For both substrate types, ‘off feature’ cells can be reduced to 0 with BSA blocking, showing the protein’s highly effective anti-adhesion properties. Regardless of blocking, the overall number of adhered cells is much higher on GO than on the silanized glass (660 on GO vs. 132 cells on silanized glass without blocking; 915 on GO vs. 220 on silanized glass with blocking). This indicates that the GO coating is better for attachment compared to the silanized glass, as further demonstrated in the number of ‘off feature’ adhered cells on the non-blocked surfaces (183 cells on GO vs. 37 cells on silanized glass).

To determine the performance of GO as substrate for single-cell arraying, the number of features carrying single cells is of particular interest. Therefore, the array features of single-cell occupancy on patterned GO and the silanized glass substrates were compared. The number of features with a single cell attached was higher on GO as compared to the silanized glass substrates, both without blocking (GO: 222 vs silanized glass: 63) and with blocking (GO: 255 vs silanized glass: 103). When evaluating the ratio of single-cell occupied to unoccupied features, it is higher for the GO coatings in all cases (0.65 non-blocked and 1.02 blocked for GO, compared to 0.10 non-blocked to 0.19 blocked on silanized glass). When comparing the ratio of single-cell occupied to unoccupied + occupied by more than one cell features, a similar image appears (0.49 non-blocked and 0.64 blocked for GO, compared to 0.10 non-blocked to 0.18 blocked on silanized glass).

## Discussion

We demonstrate a simple yet effective generation of protein microarrays on GO coatings through a direct write method in form of microchannel cantilever spotting (µCS). Substrate silanization improves GO adhesion, allowing GO flakes to form a dense coating on which subsequent protein spotting, washing and cell incubation steps for biological experiments are possible. The enhanced GO coating retention from GPTMS as intermediate layer between substrate and coating probably originates through silane and epoxy functional group interaction with hydroxyl groups available on GO flakes^22,23^. The protein microarrays are stable in lab conditions and allow selective adhesion of cells into arbitrary patterns desired e.g. for screening application or cell communication studies^2,6^. Our results underline GO as an attractive substrate material in particular for single-cell arraying. GO shows highly enhanced performance over the silanized glass control samples in regard to single-cell feature occupation (about 5 to 6 times higher ratio for single-cell carrying to unoccupied features, and a 4 to 5 times higher ration for single-cell to ‘more than one cell’ carrying features depending on blocking with BSA). Also the total number of cells attached to the array suggest that the GO coatings bind a higher amount of protein in each feature and preserve protein function better than the controls due to a more favorable interaction^24,25^. While the protein structure might still be changed by immobilization, at least in some parts of the protein^26^, studies show that in particular GO can retain a protein in a functional state (e.g. for BSA preserving AB-recognizable epitopes on GO while denaturing when in contact with reduced GO (rGO)^27^, or RGD sites on fibronection that are hypothesized to either being exposed (on GO) or buried and inaccessible for cells (on rGO) leading to different stem cell fates^28^). These observations support the use of GO coating in protein based biosensors and for the preservation of protein structure in characterization of viral particles^29^. In comparison to microwell-based single-cell arraying (also with self-assembly from solution), this approach reaches similar distribution of single- and few-cell occupancy without any topographical pre-structuring^30^ thus being a competitive option. Keeping in mind the good overall biocompatibility of GO^31^, in particular also in regard to fibroblast viability and proliferation^32^, and relative ease of the fabrication steps (spin coating and spotting of protein) makes this approach an attractive choice for cell patterning. It also allows the use of diverse substrate materials (e.g. if certain mechanical or optical properties are desired for other reasons) by providing an interface between the substrate material and the cell array without altering the bulk properties of the substrate. The selective cell adhesion in combination with the relative free choice of substrate material could also be leveraged to build cell capture devices for fishing cells out of biopsies or bodily fluids as in similar approaches towards circulating tumor cells^33,34^ or immune cells^35^. However, unspecific interactions between the adhesion proteins and other proteins in bodily fluids as well as the built up of protein-corona-like sites on the GO needs to be elucidated for specific systems for potential implications on specificity and selectivity^36,37^.

Furthermore, cell-type dependent interaction strengths of GO as e.g. demonstrated for neural cells with graphene quantum dots^38^ could be also exploited in selective cell capture and synergistically enhanced by tuning the adhesion properties locally by additional protein decoration as demonstrated in the present work.

In summary, compared to the silanized glass control patterned with protein microarrays, GO coatings offer an overall increase in the number of cells adhered and improved single-cell occupancy on array features, while retaining substrate transparency for ease of microscopy imaging. Overall, the favorable performance of the GO coatings prepared through a simple preparation method involving spin coating onto silanized surfaces, coupled with a rapid, micro-precise spotting technique, makes it an attractive alternative material for substrate coating in single-cell experiments.

## Methods

### Preparation of GO

GO dispersion was prepared by a modified Hummers’ method^39^ followed by exfoliation and purification. Briefly, graphite flakes of 50 mesh (1 g) and NaNO_3_ (0.9 g) were mixed in concentrated H_2_SO_4_ (35 mL) and left overnight to intercalate. The mixture was cooled down in an ice bath before slowly adding 4.5 g KMnO_4_ while continuously stirring. The mixture was left for 5 days at room temperature to allow graphitic oxidation. The brown slurry was diluted by slowly adding 5% H_2_SO_4_ solution (100 mL), then diluted again with 100 mL mixture of 3% H_2_SO_4_ and 0.5% H_2_O_2_. The homogenisation and complete exfoliation of graphite oxide was carried out using a vertical stirrer at a low speed for $\sim$ 1 h. The final GO dispersion was washed by repeated centrifugation and dilution with diluted H_2_SO_4_, followed by DI water, until the pH was close to neutral. The characterisation of GO is described in the electronic supplementary information S1.

### Preparation of silanized glass substrates

GPTMS coated glass substrates were prepared by cleaning glass coverslips (diameter 15 mm) in chloroform, isopropanol and water in a sonication bath for 5 min, respectively. The coverslips were dried by N_2_ and activated by oxygen plasma treatment (0.2 mbar, 100% O_2_, 100 W, 2 min, Atto plasma cleaner, Diener electronic, Ebhausen, Germany). Following this, the coverslips were placed into 1% v/v GPTMS [(3-Glycidyloxypropyl) trimethoxysilane] Toluene solution for 4 h. The silanized glass was washed with deionized (DI) water, then acetone and finally dried with N_2_. Coverslips used as control substrates were stored in a desiccator prior to experiments. All chemicals were purchased from Sigma Aldrich, Germany.

### Preparation of GO coatings

Coverslips were cleaned and treated with GPTMS as described earlier. Then, 20 µL of GO dispersion (2 mg mL^−1^ in DI water) was added to the coverslip and spin coated (4000 rpm, 1 min). GO coated substrates were stored in a desiccator prior to experiments.

### Atomic force microscopy (AFM)

All measurements were done on a Dimension Icon setup (Bruker, Germany) in tapping mode equipped with cantilevers of nominal resonance frequency of 325 kHz and a nominal force constant of 40 N/m (HQ:NSC15/Al BS, MikroMasch, NanoAndMore, Germany). For measuring the contact angle of the microdroplets on GO and SiO_x_, 3 profile lines of droplets on each sample were averaged together.

### Printing procedure

Fluorescently labelled fibronectin (with CF488A, Abs/Em Maxima: 490/515 nm, Biotium, USA) was used for printing the protein patterns on the samples. Briefly, labelled fibronectin was dissolved with DI water for a final concentration of 2 mg mL^−1^ and mixed with 20% glycerol, producing the printing ink. All patterning was carried out using an NLP 2000 instrument (Nanoink Inc., USA) under humidity‐controlled conditions via the µCS procedure, using a surface patterning tool (SPT) specialized probe^40^ (SPT-S-C10S, Bioforce Nanoscience). Probes were cleaned with oxygen plasma treatment (0.2 mbar, 100% O_2_, 100 W, 2 min, Atto plasma cleaner, Diener electronic, Ebhausen, Germany) prior to each experiment. Following cleaning, the probes reservoir was filled with 0.3–0.5 μL of the ink formulation. Optimization of the spotting procedure was carried out with variations in dwell time (0.1 and 1 s) by in-built software on the printing setup. Optimized experiments used a dwell time of 1 s (otherwise stated), with 30–50% relative humidity. Protein patterns were produced with 10 × 10 droplet array and 30 μm XY spacing between each droplet. For cell adhesion experiments, samples were printed in a 3 × 3 array with a 30 µm XY spacing, which was repeating to produce a 9 × 9 array with a spacing of 90 µm (total of 81 arrays or 729 droplets). Printed samples were maintained in a desiccator for minimum 48 h prior to cell exposure.

### Fluorescence microscopy

Ink patterns were observed under the fluorescence microscope (Eclipse 80i, Nikon). The maximum fluorescence and average droplet area per pattern was assessed by the in-built software (NIS element, Nikon, Germany) and ImageJ. GO coatings were additionally visualized under polarized light conditions (Sarfus-HR microscope, Nanolane).

### Antibody incubation

Fibronectin functionality was evaluated through antibody binding. Printed GO and control substrates were blocked with 5% (v/v) BSA (Sigma-Aldrich) in PBS solution at room temperature (20 min). The blocking solution was removed and the monoclonal mouse anti-fibronectin antibody in PBS (10 μg/ml, abcam) was added. The samples were incubated at room temperature for 90 min. Following incubation, samples were washed 3 times with PBS and subsequently exposed to the anti-mouse antibody-Cy5 conjugates (7.5 μg/ml, λ_*Exc*_ = 649 nm, λ_*Em*_ = 666 nm, Invitrogen) at room temperature in dark conditions for 60 min. Samples were washed twice with PBS before being stored at 4 °C and submerged in PBS. The secondary antibody position was assessed using fluorescent microscopy.

### Cell adhesion assays

NIH-3T3 cell line (Cell Technology) was used to further validate the functionality of printed fibronectin and effect on single-cell adhesion. Cells were cultured in Dulbecco's modified Eagle's medium (DMEM, Life Technologies, Germany) supplemented with 15% fetal bovine serum (FBS, Sigma‐Aldrich, Germany) for overnight under standard cell‐culture conditions: 37 °C and 5% CO_2_. The cells were washed with PBS to remove culture medium and detached by prewarmed (37 °C) 600 µl Tripsin-EDTA (Thermo-Fisher scientific, Germany) for 5 min. The cells were collected in an Eppendorf microcentrifuge tube and centrifuged for 5 min with 5 × 1000 rpm. The cells were suspended in DMEM with 10% FBS. Prior to cell exposure, the printed substrates were washed with PBS and blocked with 10% v/v BSA in PBS for 20 min at 37 °C. For cell seeding, 1 × 10^7^/mL cells (50 µl) were exposed to blocked samples (for unblocked samples directly after washing with PBS) for 30 min or 1 h at 37 °C. Following 30-min cell exposure, samples were washed with PBS and fixed with 4% v/v paraformaldehyde in PBS for 20 min at room temperature. To stain the cell nuclei samples were washed with PBS and incubated with 4′,6-diamidino-2-phenylindole (DAPI) (1 µg/ml, 5 min) at room temperature. After the washing step with PBS, fluorescence microscopy was performed to visualize the fibronectin patterns in green (GFP channel) and the cells nuclei in blue (DAPI channel).

### Cell counting

Cells were counted from the merged images taken by fluorescence microscope using in-built software of microscope (NIS element, Nikon, Germany). The counting of individual cells was done manually, with cells on the edges excluded. Cells were counted on high-magnification images and then these subsets were extrapolated to the overall patterned area.

## Supplementary Information

Supplementary Information.
