# Supplementary material for: Protein spot arrays on graphene oxide coatings for efficient single-cell capture
Source: Sci Rep. 2022 Mar 10;12:3895. doi: 10.1038/s41598-022-06225-4 (PMC8913813; doi:10.1038/s41598-022-06225-4)
Supplement: Supplementary file 1 — Supplementary Information. [file 41598_2022_6225_MOESM1_ESM.pdf]

Supplementary Information for

Protein spot arrays on graphene oxide coatings for efficient single-cell capture

R. Kumar,[1†] S. Llewellyn,[2,3†] S. K. Vasantham,[1] K. Nie,[2] S. Sekula-Neuner,[4] A. Vijayaraghavan,[2]* M. Hirtz[1]*

[1]Institute of Nanotechnology (INT) and Karlsruhe Nano Micro Facility (KNMF), Karlsruhe Institute of Technology (KIT), Karlsruhe, Germany. [2]Department of Materials, The University of Manchester, Manchester, UK. [3]Blond McIndoe Laboratories, Faculty of Biology Medicine and Health, University of Manchester, Manchester, UK. [4]n.able GmbH, Eggenstein-Leopoldshafen, Germany.*Corresponding authors.

Email: aravind@manchester.ac.uk (A.V.); michael.hirtz@kit.edu (M.H.)

[†]These authors contributed equally to this work

Graphene Oxide Flake Characterization

The physical and chemical characteristics of the graphene oxide (GO) flakes was analysed prior to substrate preparation. GO absorbance was measured (200-800 nm wavelength) using double distilled water (ddH$_2$O) as a baseline (Genesys 10S UV-Vis Spectrophotometer, ThermoScientific). 2 mg/ml of GO solution was diluted in ddH$_2$O for further measurement concentrations of 0.02 mg/ml and 0.5 mg/ml. 0.5 mg/ml and 2 mg/ml of GO solutions were spin coated on cleaned Si/SiO2 substrates for flake imaging and Raman spectroscopy respectively. The lateral flake size was measured using SEM images (Zeiss Ultra; Accelerating Voltage; 10 kV). A minimum of 10 photos were taken with 478 individual GO flakes counted in total. Evaluation of lateral size was achieved using the measuring parameters as set out in the National Physical laboratory graphene manual.[1] The Raman spectra of the GO solution was acquired using a 514 nm Raman microscope laser, set at power <15 μW (RM-1000 System, Renishaw). A total of 3 measurements was done. GO spectra and G and D peaks were processed and fitted respectively using WiRE™ software (Renishaw). The chemical composition of GO flakes was evaluated using XPS (Kratos Axis Ultra Hybrid). GO solution (2 mg/ml) was drop casted onto cleaned Si/SiO$_2$ substrate and dried in vacuum for minimum 48 hours prior to measurements. A monochromatic Al Kα source (1486.6 eV, 10 mA emission at 150 W, spot size 300 × 700 μm) was used for analysis. The oxidation data and peak fitting for carbon 1s was processed using CasaXPS software. Prior to peak fitting, a Shirley Background was set and Carbon peak calibrated at 284.5 eV. The GO solution was loaded into a Zetasizer (Malvern Zetasizer) for zeta potential measurements. Three measurements were taken in total from three separately prepared

[1] A. J. Pollard, K. R. Patton, C. A. Clifford, E. Legge, A. Oikonomou, S. Haigh, C. Casiraghi, L. Nguyen, D. Kelly, in *Characterisation Struct. Graphene*, National Physical Laboratory, Middlesex, **2017**, pp. 52–55.

GO solution (0.02 mg/ml). The machine was thermally equilibrated at 25 °C for 2 minutes prior to each measurement.

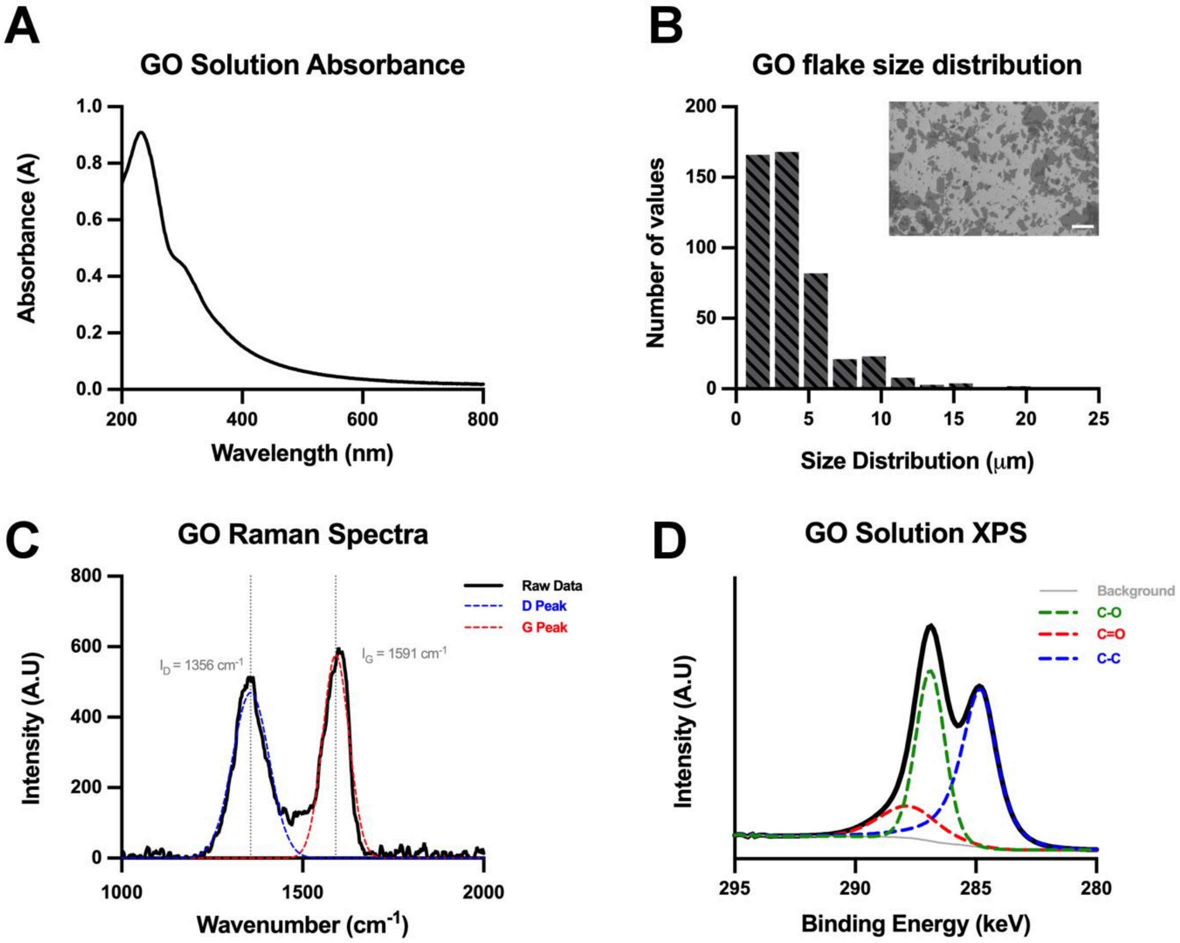

Figure S1 | Characterization of the graphene oxide flakes. (A) UV-Vis absorbance of diluted GO solution (0.02 mg ml-1). **(B)** GO flake size. Mean flake size calculated 3.60 ± 2.95 µm. Graph inset includes representative SEM image of GO flakes. Scale bar set at 20 µm. **(C)** Raman spectra of GO solution, with D & G peak annotated. ID/IG = 0.88. XPS Spectra of GO with characteristic sp3 (C-C), C-O and C=O peak fittings. Carbon:Oxygen ratio calculated at 2.5. Table S1 outlines characteristic chemical and physical values of GO solution used during this work.

Table S1. Physical and chemical characteristics of GO solution.

Mean size (μm)	ζ-potential (mV)	I_D/I_G	C/O
3.60 ± 2.94	-24.1 ± 1.95	0.88	2.5

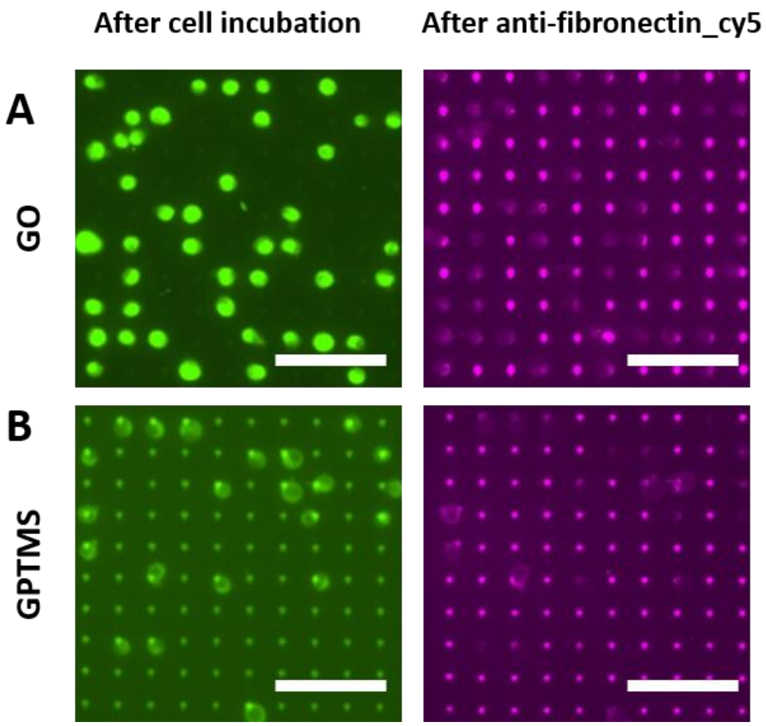

Figure S2 | Detection of fibronectin pattern by anti-fibronectin antibody. After 3T3 cells were incubated on both samples, (**A**) GO and (**B**) GPTMS coating, the fibronectin pattern was visualized by anti-fibronectin antibody labelled with Cy-5 fluorescent dye. The 3T3 cells are visible in the green channel as bigger round spots over the fibronectin array by autofluorescence. Scale bars equal 100 μm.

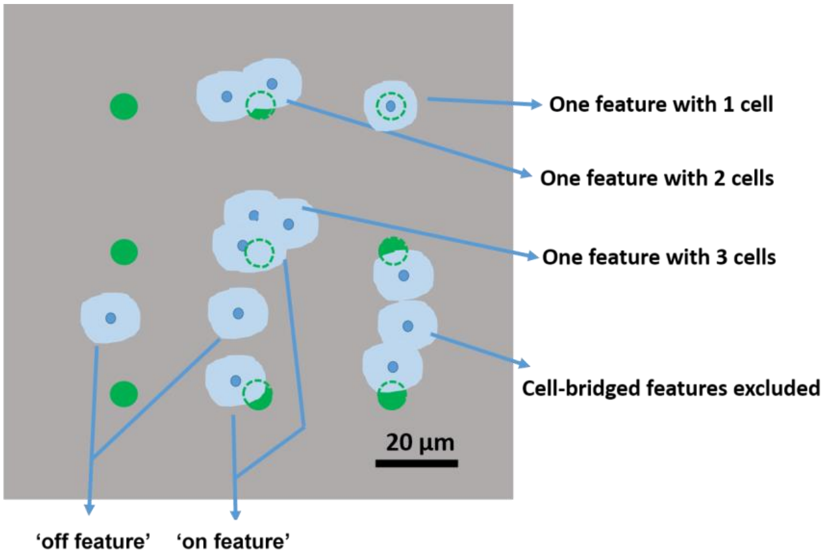

Figure S3 | Scheme on definitions of cell counts. The scheme depicts the different cell distributions observed on samples and how these were counted for the cell occupancy statistics. Cells or few-cell clusters attached to an array feature (green dots) were defined as 'on feature' and counted according to cell number. Cells not attached to any feature but being found in the patterned area (i.e. in between array features) were defined and counted as 'off feature'. Cell clusters bridging between array features were excluded from counting.

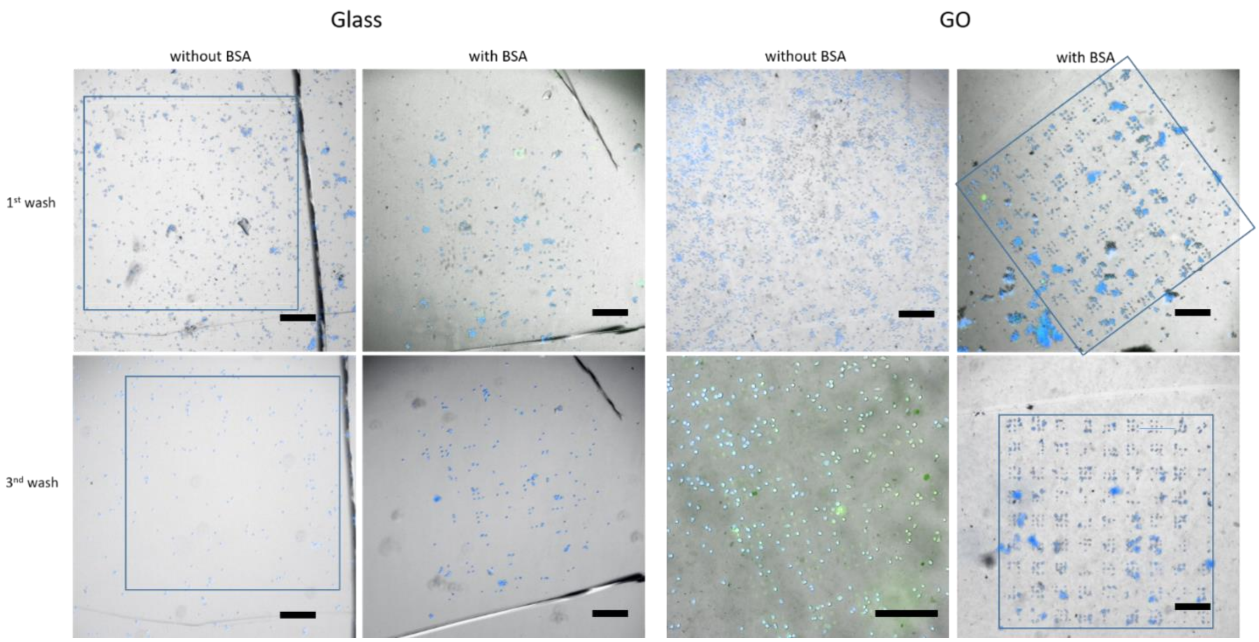

Figure S4 | Cell adhesion on micropatterns after repeated washing. The figure shows exemplary microscopy images (bright field and fluorescence channel merged) for GPTMS coated glass (left) and GO coated glass (right), with and without BSA blocking after the first wash (upper line) and after third wash (lower line). Scale bars equal 200 µm.

Table S2. Quantification of cell adhesion on micropatterns after subsequent washing steps. Quantification of the numbers of the cells adhered onto the GO coating and silanized glass substrates with and without prior BSA blocking after first and third washing.

**Substrate type**	**Blocking**	**Number of cells**			
		**on feature**		**off feature**	
		**1st wash**	**3rd wash**	**1st wash**	**3rd wash**
GO	-	3186	477	-*	183
	BSA	1062	915	36	0
Glass	-	1011	95	-*	37
	BSA	253	220	46	0

*not counted, as of too high cell-density
